# Cross-national disparities in drug interaction reporting and assessment between Switzerland and Austria: A retrospective, registry-based study using pharmacovigilance data

**DOI:** 10.1371/journal.pone.0356774

**Published:** 2026-08-21

**Authors:** Marcel Rainer, Adrian Martinez De la Torre, Romuald Bellmann, Andrea Michelle Burden, Stefan Weiler

**Affiliations:** 1 Institute of Pharmaceutical Sciences, ETH Zurich, Zurich, Switzerland; 2 Hospital Pharmacy, Department of Medical Services, Kantonsspital Baden AG, Baden, Switzerland; 3 Faculty of Health Sciences and Medicine, University of Lucerne, Lucerne, Switzerland; 4 Swiss Paraplegic Research, Nottwil, Switzerland; 5 Clinical Pharmacokinetics Unit, Division of Intensive Care and Emergency Medicine, Department of Internal Medicine I, Medical University of Innsbruck, Innsbruck, Austria; 6 Division of Clinical Immunology and Rheumatology, University of Alabama at Birmingham, Birmingham, Alabama, United States of America; 7 Institute of Primary Care, University of Zurich, Zurich, Switzerland; Universiti Sains Malaysia, MALAYSIA

## Abstract

Drug–drug interactions (DDIs) are a significant contributor to adverse drug reactions (ADRs) in clinical practice. Despite established pharmacovigilance systems, underreporting and inconsistent causality assessment remain challenges. Switzerland and Austria offer a unique comparative setting due to similar healthcare infrastructures but different pharmacovigilance system designs. This study aims to compare pharmacovigilance system outcomes of Switzerland and Austria by examining how system designs may influence DDI reporting patterns and report quality. We retrospectively analysed 1,499 individual case safety reports involving DDIs submitted between January 2008 and April 2024 to the national pharmacovigilance systems of Switzerland (n = 1,159) and Austria (n = 340). As indicators of system performance, reports were assessed for demographics, reporter categories, seriousness of outcomes in relation to polypharmacy (expressed as reporting odds ratios [RORs] and confidence intervals [CIs]), ADR types, and pharmacological plausibility. Cross-national disparities were evident: Switzerland reported 3.4 times more DDI cases than Austria (mean 5.8 vs. 1.7 cases/month), with comparable patient demographics and differing reporter proportions. Most reports were classified as serious (80% Switzerland, 84% Austria), with polypharmacy strongly associated with serious outcomes in both countries (Switzerland: ROR 3.71, 95% CI 3.26–4.23, p < 0.0001; Austria: ROR 1.98, 95% CI 1.60–2.44, p < 0.0001). Reported ADRs differed qualitatively, with Switzerland predominantly reporting mechanisms whereas Austria reported more frequently symptoms. Differences were also found in the pharmacological consistency between ADR and associated drugs. These cross-national disparities in DDI reporting volume, content, and quality suggest that pharmacovigilance system design and specialist involvement influence reporting. The integration of clinical pharmacologists and pharmacists in ADR processing warrants further investigation, given its potential to strengthen DDI-related pharmacovigilance practices. These findings provide actionable insights for policymakers and regulatory agencies seeking to strengthen their pharmacovigilance systems through strategic specialist integration.

## Introduction

Drug interactions are highly prevalent in clinical practice [1] and are associated with significant health risks and economic burdens, many of which are preventable [2,3]. Despite their clinical significance, drug interactions often remain under-recognized in routine care [4,5]. This underestimation is intensified by several additional factors: an aging population, increasing polypharmacy, the growing complexity of treatment regimens, and persistent gaps in knowledge regarding pharmacokinetics and pharmacodynamics [1,6]. Together, these factors increase the complexity in prescribing and monitoring, potentially elevating the risk of adverse drug reactions (ADRs).

Pharmacovigilance systems are essential for mitigating these risks by enabling detection, assessment, and prevention of ADRs outside the controlled environments of randomized controlled trials. These systems serve as tools for identifying drug-related problems in real-world settings, guiding regulatory decisions, informing clinical guidelines, and improving prescribing practices [7]. Yet, spontaneous reporting lacks a defined denominator and is prone to underreporting and prescriber-level biases, complicating signal detection for drug interactions [8]. In a study by Beeler and colleagues, a substantial proportion of ADR-related hospitalizations went unreported to regulatory authorities despite mandatory reporting requirements [9]. Given these limitations, cross-national comparisons of comparable healthcare systems and medication use may provide critical insights into how system design influences reporting and data quality, which are essential for the accurate interpretation of drug safety events.

Switzerland and Austria are neighbouring European countries with comparable population sizes (approximately 9 and 9.2 million inhabitants, respectively) [10], age distributions [10], universal healthcare coverage [11], and access to largely overlapping pharmaceutical markets. These similarities provide a useful basis for comparison, while differences in pharmacovigilance organisation and reporting structures exist. In Switzerland, the reporting system is managed centrally through the Electronic Vigilance System (ELViS) [12] with reports assessed at clinical pharmacology units. Whereas Austria relies on its Federal Office for Safety in Health Care (BASG) [13] to oversee and assess pharmacovigilance activities without mandatory involvement of clinical pharmacology specialists in routine report assessments. These systems differ in the organization of pharmacovigilance activities, reporting workflows, and expert involvement, which may contribute to variations in drug interaction reporting and clinical assessment quality.

This study aims to investigate drug interaction reporting patterns and report quality across different age groups in Switzerland and Austria, exploring the potential role of differences in pharmacovigilance system design. By identifying cross-national differences, we seek to improve the understanding of how system structure may impact the recognition of drug interactions, and ultimately informing strategies to enhance patient safety through strengthening pharmacovigilance practices.

## Methods

### Study design and reporting

We conducted a cross-sectional, retrospective, observational study examining drug interaction reporting patterns in Switzerland and Austria. Both quantitative and qualitative analytical approaches were used as system performance indicators. The study is reported in line with the Strengthening the Reporting of Observational Studies in Epidemiology (STROBE) guidelines for cross-sectional studies (version 4) [14]. The checklist is provided in the Supplementary Material (S1 Table). The electronic forms for notification of ADRs provided by the competent authorities are similar in both countries [12,13]. Their major components are outlined in the Supplementary Material (S2 Table).

### Data sources and extraction

We extracted pharmacovigilance data from VigiBase^®^, the World Health Organisation's global database of ADRs, on 3 April 2024. VigiBase^®^, managed by the Uppsala Monitoring Centre, has collected individual case safety reports (ICSRs) from over 140 countries since 1968 [15]. We included all ICSRs reported under the Medical Dictionary for Regulatory Activities (MedDRA^®^) version 26.1 higher-level term “Drug Interaction” [16], spanning from the first drug interaction report in both countries to the extraction date, 1 January 2008 and 1 April 2024. Subcategories by preferred terms were used to differentiate interaction types (e.g., drug-drug, drug-herb, drug-alcohol), with multiple interaction types possible within a single case. The completeness score with a maximum of 1 was used to measure the amount of clinically relevant information in an ICSR and to assess the quality of documentation. Data fields available in the electronic notification forms of each country's pharmacovigilance system were summarised and guided variable selection.

### Study population and variables

Cases were stratified by age into three groups: children (<18 years), adults (18–64 years), and elderly (>64 years). We included cases with reported patient ages below 120 years as a plausibility criterion after deduplication. Primary variables included the frequency of drug interaction reports, patient demographics, interaction types, and reported outcomes. We assessed case seriousness and categorized reasons for serious classification including hospitalization, life-threatening conditions, and fatalities. Drug classifications were analysed using the Anatomical-Therapeutic-Chemical (ATC) coding system.

### Statistical analysis

We conducted descriptive analyses using R statistical software (version 4.2.2). For categorical variables, we calculated frequencies and proportions. For continuous variables, we computed means or medians with standard deviations (SDs) or interquartile ranges (IQRs), as appropriate for the data distribution. We calculated reporting odds ratios (ROR) with 95% confidence intervals (CIs) to assess associations between polypharmacy (defined as ≥5 drugs co-reported) and serious outcomes. Statistical significance was assessed using a Wald test (p < 0.05), with polypharmacy as the dependent variable and case seriousness as the predictor. Comedications were explored by constructing undirected drug-drug interaction (DDI) networks from ICSRs by identifying all co-reported drug pairs within each case in secondary analyses. For each drug, connectivity metrics (node degree as the number of unique co-medication partners and weighted strength based on edge counts) were calculated and visualized using bar plots with summary tables. Time series analysis examined reporting patterns, including mean reports per year, SDs, ranges, and coefficient of variances (CVs) to allow a comparison of variability between countries with different mean case counts. Rolling standard deviations (rSDs) were computed to identify temporal fluctuations in reporting patterns. We analysed ADR-drug associations using co-occurrence frequencies, visualized through heatmaps.

### Ethics

The study was conducted in accordance with the Declaration of Helsinki. This study used exclusively anonymized secondary data from VigiBase, the WHO global database of individual case safety reports. Following consultation with the Cantonal Ethics Committee of Zurich, ethics committee approval was not required because the study falls outside the scope of the Swiss Federal Act on Research involving Human Beings (Human Research Act, HRA; Article 2), which does not apply to research involving anonymously collected or anonymized health-related data. Reports are anonymised in line with WHO and Uppsala Monitoring Centre (UMC) guidelines; no identifiable patient information is available and patient informed consent was not required. The probability of a causal relationship between the drug and reported event varies between cases and does not reflect the views of the UMC or WHO.

## Results

### Study population, demographics, and reporters

**Fig 1:**
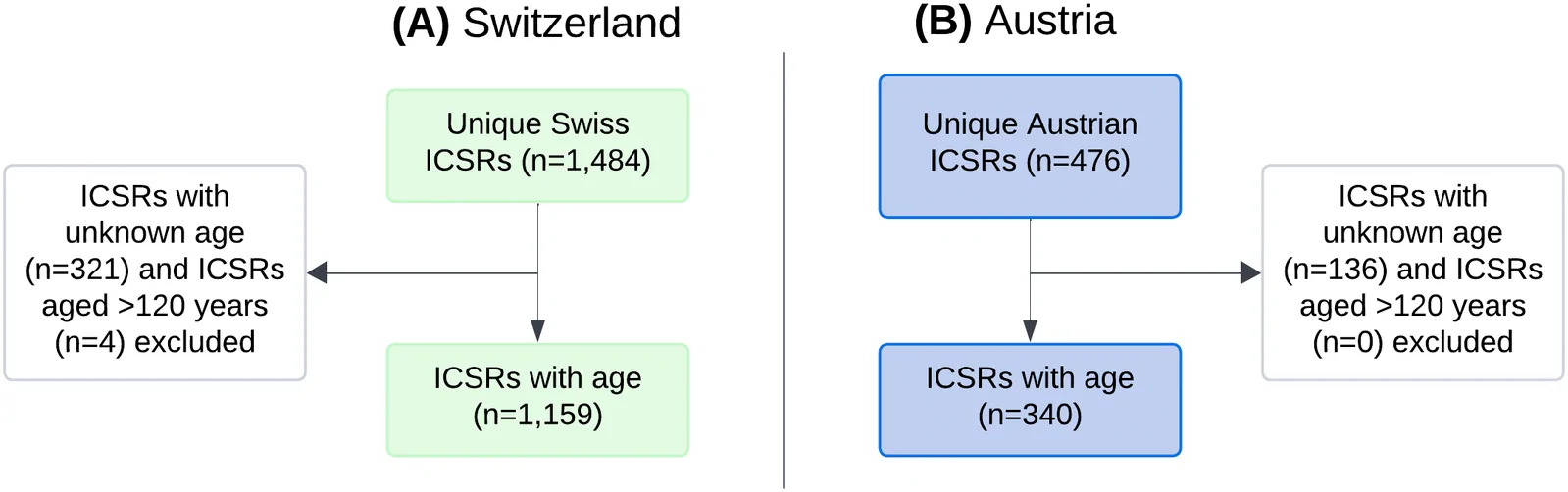
Flowchart of extracted individual case safety reports (ICSRs) from the pharmacovigilance database (WHO VigiBase^®^) for: (A) Switzerland and (B) Austria, including reasons for exclusion (duplicates, implausible or missing age).

A total of 1,960 unique cases of DDIs reported between 1 January 2008 and 17 April 2024 in Switzerland and Austria were included. After excluding 461 cases (23.5%) due to unknown age or aged >120 years, the final dataset comprised 1,159 reports from Switzerland (Fig 1A) and 340 from Austria (Fig 1B). The data completeness, using standardized completeness scores, was comparable between the two countries: in Switzerland the mean score was 0.471 (± 0.229) and in Austria 0.433 (± 0.215). Table 1 summarizes the characteristics of the study population, reporters, case seriousness, and drug interaction type, stratified by the predefined age groups and country.

**Table 1:** Demographic characteristics, reporters, case seriousness, and interaction types of drug interaction reports, extracted on 3 April 2024. Age stratification was performed as follows: adults aged 18–64 years, children aged <18 years, and the elderly aged >64 years.

	Switzerland			Austria		
	Adults	Elderly	Children	Adults	Elderly	Children
Characteristics	n = 589	n = 526	n = 44	n = 190	n = 133	n = 17
Age, median (IQR)	49 (12)	77 (7)	10 (6)	43 (14)	11 (6)	7 (7)
Sex						
Female, n (%)	266 (45.2%)	254 (48.3%)	9 (20.5%)	92 (48.4%)	72 (54.1%)	6 (35.3%)
Male, n (%)	317 (53.8%)	267 (50.8%)	18 (40.9%)	97 (51.1%)	59 (44.4%)	10 (58.8%)
Missing, n (%)	6 (1.0%)	6 (1.1%)	17 (38.6%)	1 (0.5%)	2 (1.5%)	1 (5.9%)
Reporter*						
Physician, n (%)	392 (66.6%)	367 (69.2%)	21 (47.7%)	124 (65.3%)	82 (61.7%)	7 (41.2%)
Pharmacist, n (%)	47 (8.0%)	69 (13.0%)	4 (9.1%)	7 (3.7%)	14 (10.5%)	2 (11.8%)
Consumer, n (%)	37 (6.3%)	22 (4.2%)	4 (9.1%)	31 (16.3%)	32 (24.1%)	3 (17.6%)
Other, n (%)	141 (23.9%)	96 (18.3%)	16 (36.4%)	41 (21.6%)	27 (20.3%)	5 (29.4%)
Missing, n	5 (0.8%)	0 (0%)	0 (0%)	2 (1.1%)	2 (1.5%)	0 (0%)
Serious, n (%)	461 (78%)	472 (89%)	39 (89%)	152 (81%)	106 (80%)	11 (65%)
Missing, n (%)	0 (0%)	0 (0%)	0 (0%)	3 (1.6%)	1 (0.8%)	0 (0%)
Outcome						
Death, n (%)	16 (2.7%)	32 (6.0%)	12 (27.3%)	10 (5.3%)	17 (12.8%)	0 (0%)
Interaction Types*						
Drug-drug interaction, n (%)	583 (99.0%)	520 (98.9%)	41 (93.2%)	189 (99.5%)	130 (97.7%)	17 (94.1%)
Drug-disease, n (%)	3 (0.5%)	0 (0%)	0 (0%)	1 (0.5%)	0 (0%)	0 (0%)
Drug-gene, n (%)	4 (0.7%)	0 (0%)	2 (4.5%)	0 (0%)	0 (0%)	1 (5.9%)
Drug-device, n (%)	2 (0.3%)	2 (0.4%)	1 (2.3%)	0 (0%)	0 (0%)	0 (0%)
Drug-food, n (%)	1 (0.2%)	1 (0.2%)	0 (0%)	5 (2.6%)	2 (1.5%)	0 (0%)
Drug-C2, n (%)	17 (2.9%)	2 (0.4%)	0 (0%)	3 (1.6%)	2 (1.5%)	0 (0%)
Drug-tobacco, n (%)	0 (0%)	1 (0.2%)	0 (0%)	1 (0.5%)	0 (0%)	0 (0%)

* Reporter categories and drug interaction types are not mutually exclusive at the case level, as a single report may document multiple reporter types and/or interaction types.

Abbreviations: C2, Alcohol; IQR, Interquartile Range; n, Number

The median age of reports from Switzerland was higher than that of Austria (64 years [IQR: 48–75] vs. 58 years [IQR: 38–72]). In both countries, adults represented the largest reported group (51% in Switzerland, 56% in Austria), followed by elderly patients (46% in Switzerland, 39% in Austria), and children (3.8% in Switzerland, 5.0% in Austria). Sex distribution was balanced, with females accounting for 45% of Swiss and 50% of Austrian cases. Within age groups, the sex distribution remained consistent for adults and elderly individuals (Table 1). However, among children, females represented only approximately one-third in both countries.

Healthcare professionals were the primary reporters of DDIs. Physicians submitted the majority of reports (67% in Switzerland, 63% in Austria), followed by pharmacists (10% in Switzerland, 7% in Austria). Specifically, the proportion of consumer-reported cases were substantially higher in Austria (20%) compared to Switzerland (5%), with similar total numbers (Austria: n = 73, Switzerland: n = 63).

### Reporting patterns and severity

**Fig 2:**
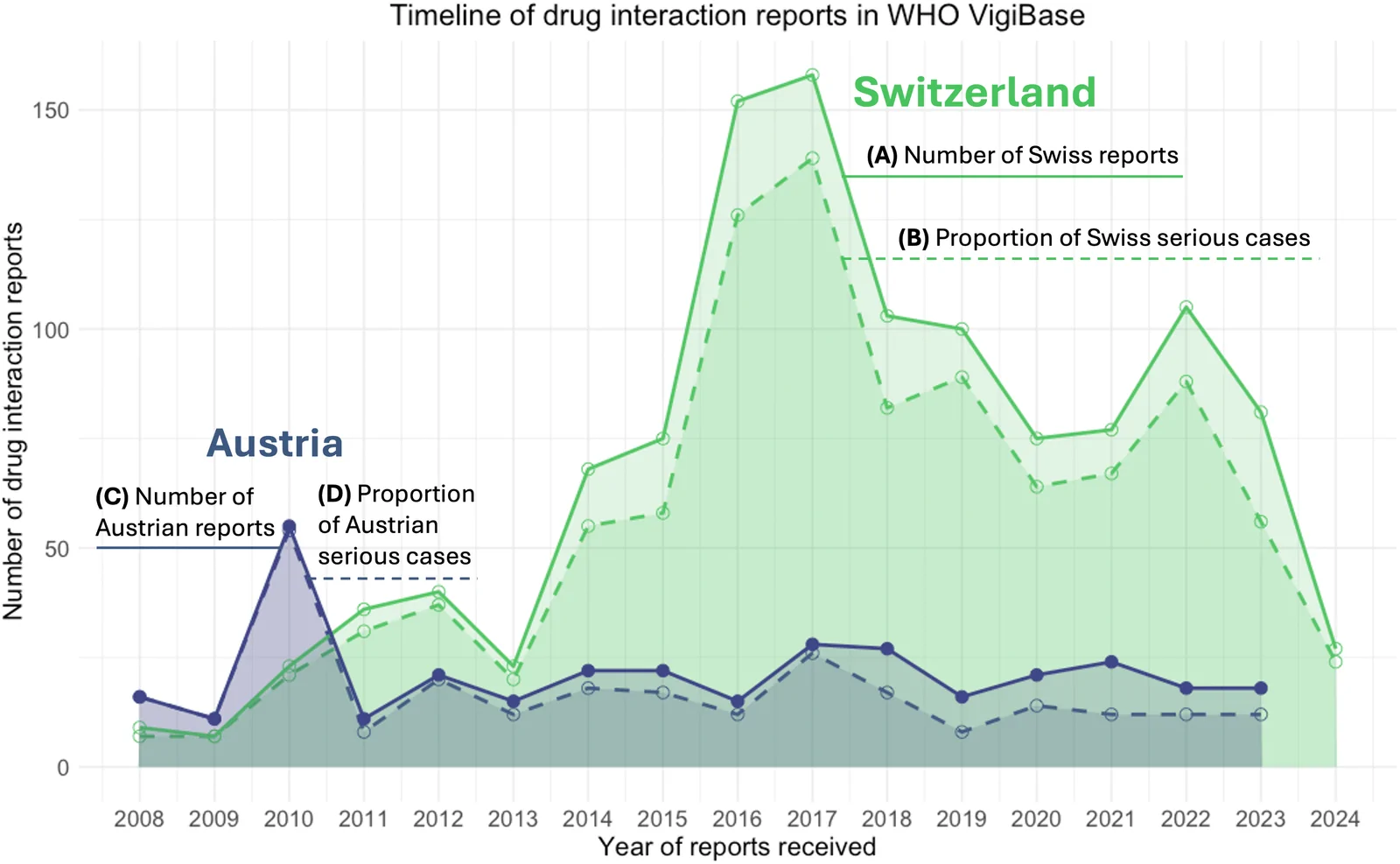
Temporal patterns of drug interaction reports submitted to the World Health Organisation's (WHO) VigiBase^®^ pharmacovigilance database from Switzerland (green) and Austria (blue). Solid lines (2A for Switzerland, 2C for Austria) represent the annual count of drug interaction reports, whereas dotted lines represent the proportion of these reports classified as serious adverse events (2B for Switzerland, 2D for Austria). The Swiss reporting pattern demonstrates marked variability over time, characterised by fluctuations and reaching its highest point in 2017. Swiss reports exceeded those from Austria as of 2011. Austrian reporting, in contrast, maintained relatively stable levels since 2011, peaking in 2010, and with no new reports documented in 2024 at the time of the extraction (3 April 2024).

Fig 2 illustrates the time trends in ICSRs over the study period (Fig 2A for Switzerland, 2C for Austria) and the proportion of cases classified as serious (Fig 2B for Switzerland, 2D for Austria). The reporting frequency showed large differences and distinct patterns between the countries. Switzerland reported 3.4-fold more cases than Austria, averaging 5.8 reports per month compared to Austria reporting 1.7 cases per month. The highest annual number of reports was observed in 2017 (n = 199) for Switzerland, and in 2010 (n = 62) for Austria. Reporting in Switzerland exhibited substantial year-to-year variability (mean: 87.1 cases, SD: 58.5, CV: 0.67, range: 7–199 cases), while in Austria a more stable reporting trend was observed (mean: 29.8 cases, SD: 11.9, CV: 0.4, range: 11–62 cases). In Switzerland, distinct temporal fluctuations were evident in 2012 (rSD 24.0 to 5.6), 2018 (rSD 7.6 to 11.2), and 2020 (rSD 8.1 to 2.0), indicating temporal shifts in reporting intensity.

Overall, the majority of reports were classified as serious in both countries, with 80% in Switzerland and 84% in Austria. The most frequent reasons for seriousness classification were hospitalisation or prolonged hospitalization (44% Switzerland, 36% Austria) and other medically important conditions (41% Switzerland, 48% Austria). Life-threatening conditions were reported in 7% and 8% of cases in Switzerland and Austria, respectively. The proportion of fatal outcomes was comparable, at 6% in reports from Switzerland and 7% from Austria. Older adults represented the majority of fatal cases (53% Switzerland, 63% Austria). In contrast, child fatalities accounted for 20% of all fatalities in Switzerland, while no child fatalities were reported in Austria throughout the study period.

### Interaction types and associated drugs

**Fig 3:**
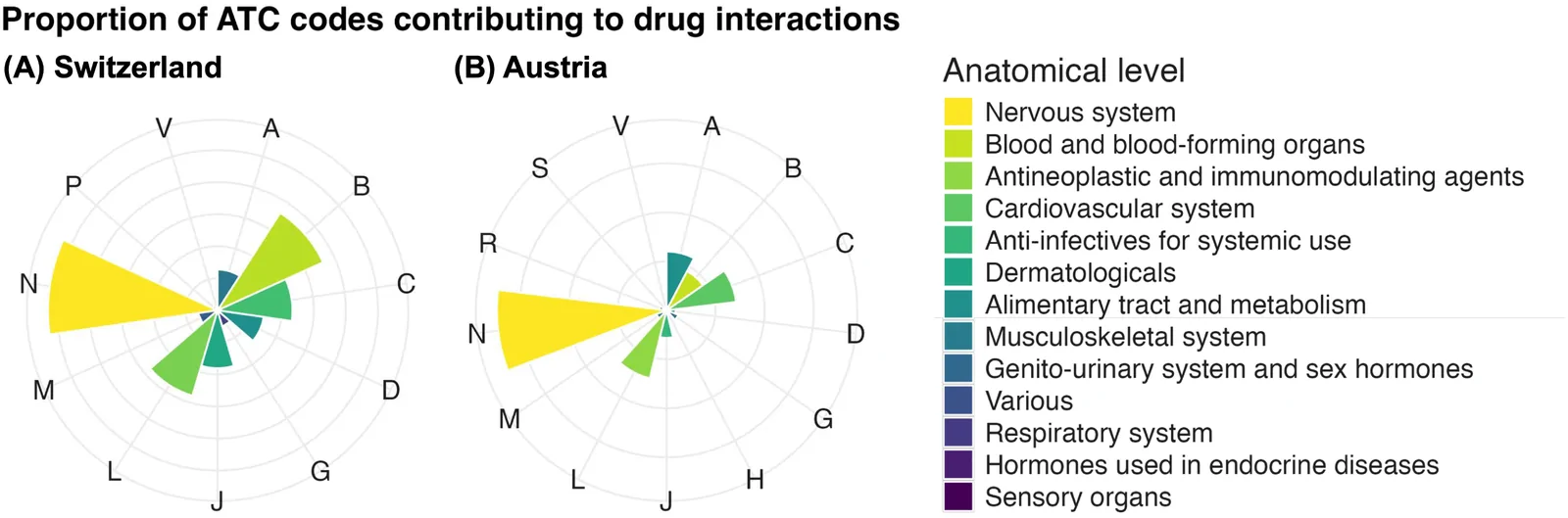
Proportional distribution of drugs implicated in drug interactions calculated by their anatomical-therapeutic-chemical (ATC) code and ordered by anatomical level among all reported cases, stratified by country. Drugs affecting the nervous system were predominantly involved in both countries, with varying proportions of other anatomical levels between countries.

The majority of reports in both countries involved DDIs, accounting for 97.4% of Swiss cases and 98.8% of Austrian cases (Table 1). Less common interaction types included drug-alcohol interactions (n = 19, 1.6% Switzerland; n = 5, 1.5% Austria), drug-gene interactions (n = 6, 0.5% Switzerland; n = 1, 0.3% Austria), drug-food interactions (n = 2, 0.2% Switzerland; n = 7, 2.1% Austria), drug-disease interactions (n = 3, 0.3% Switzerland; n = 1, 0.3% Austria), and drug-tobacco interactions (n = 1, 0.1% Switzerland; n = 1, 0.3% Austria). Drug-device interactions were only reported in Switzerland (n = 5, 0.4%). Drugs affecting the nervous system were most frequently implicated in DDIs, representing 31% of all reports in Switzerland and 36% in Austria. Fig 3 shows the proportion of involved drugs by their ATC classification system stratified by country (3A for Switzerland, 3B for Austria). Temporal trends in drug-interaction involvement are shown in the Supplementary Material (S1-S2 Figs in S1 File).

In total, 9’520 drugs were reported in Switzerland, with the number of drugs per case ranging from 1 to 17 drugs across the 5^th^ to 95^th^ percentiles (mean 6.4, SD 5.6). In Austria, 5’043 drugs were reported, with a distribution from 1.75 to 15 drugs per case in the 5^th^ to 95^th^ percentiles (mean 5.6, SD 5.2). The presence of polypharmacy was significantly associated with an increased risk of a serious outcome. In Switzerland, the ROR for serious outcomes involving polypharmacy was 3.71 (95%-CI 3.26–4.23, p < 0.0001), while in Austria it was 1.98 (95%-CI 1.60–2.44, p < 0.0001). The case-based proportion of polypharmacy over the study period is illustrated in the Supplementary Material (S3-S6 Figs in S2 File). The specific drugs most commonly involved in reported interactions varied between both countries. In Switzerland, the top interacting agents included acetylsalicylic acid, amiodarone, rivaroxaban, phenprocoumon, and tramadol. In contrast, the most frequently implicated drugs in Austria were acetylsalicylic acid, pantoprazole, bisoprolol, trazodone, and risperidone. Network analysis revealed country-specific comedication patterns, with Austrian cases showing higher connectivity than Swiss cases, reflecting more complex polypharmacy. The most frequently co-reported drugs also exhibited high network connectivity in Austria (100%; acetylsalicylic acid, pantoprazole, bisoprolol, and risperidone) and in a proportion for Switzerland (33%; amiodarone, rivaroxaban, phenprocoumon). Network metrics are detailed in the Supplementary Material (S7 Fig).

### Adverse drug reactions

Notable differences were observed in the type of reported ADRs in Switzerland and Austria. Swiss reports predominantly described mechanistic or pharmacologically expected outcomes, such as acute kidney injury (5.8%), QT interval prolongation (3.7%), drug ineffectiveness (3.2%), and increased drug levels (3.0%). In contrast, Austrian reports more frequently documented symptomatic manifestations, such as delirium (1.0%), agitation (0.9%), dizziness (0.8%), and chronic obstructive pulmonary disease (0.8%).

**Fig 4:**
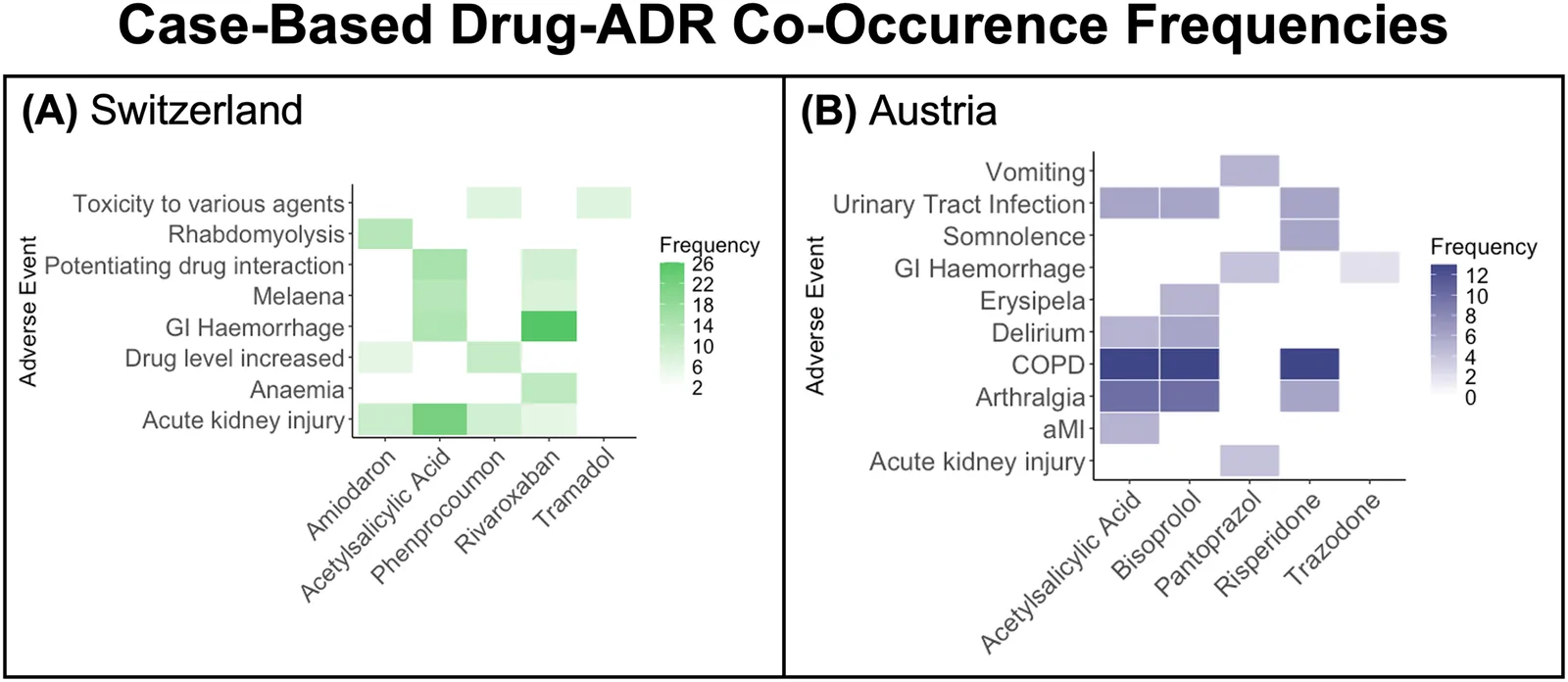
Heatmaps of drug interaction reports from Switzerland (panel 4A) and Austria (panel 4B). The plots display the most frequently reported drugs linked to the most commonly co-reported adverse drug reactions (ADRs), stratified by co-reported drugs. The colour scale of each cell reflects the number of co-occurrences, illustrating patterns of suspected drug interactions and associated ADRs. Abbreviations: ADR, Adverse Drug Reaction; aMI, Acute Myocardial Injury; COPD, Chronic Obstructive Pulmonary Disease; GI, Gastrointestinal.

The analysis of drug-ADR associations revealed notable patterns. Fig 4 presents heatmaps of drug-ADRs co-occurrence frequencies. In Swiss data (Fig 4A), reported ADRs were largely consistent with known pharmacological mechanisms. However, Austrian data (Fig 4B) revealed unexpected and inconsistent associations that diverged from established pharmacological expectations. Examples of these pharmacological inconsistencies included reports of gastrointestinal haemorrhage with pantoprazole, myocardial infarction and arthralgia with acetylsalicylic acid. Additionally, urinary tract infections were reported with acetylsalicylic acid, bisoprolol, and risperidone.

## Discussion

This study provides a descriptive analysis of drug interaction reporting in Switzerland and Austria over a 16-year period, focusing on quantitative and qualitative differences in pharmacovigilance systems, reporting patterns, demographics, types of ADRs, and drug-ADR associations. Despite comparable healthcare infrastructures and access to similar pharmacological agents, substantial disparities were observed between the two countries, reflecting a potential influence of system design and reporting culture on pharmacovigilance outcomes.

Switzerland exhibited substantially higher reporting volumes and greater variability over time, which may reflect stronger professional engagement with the Electronic Vigilance System (ElViS) [12], particularly among physicians and pharmacists. In contrast, Austria showed a lower but more stable reporting frequency, with a notably higher proportion of reports submitted by consumers. This is consistent with the recognised variability in patient engagement in pharmacovigilance practice [17]. However, the marked difference between the neighbouring countries suggests that underreporting may be more prevalent among Austrian healthcare academics, given that the absolute number of consumer reports is comparable due to the similar population sizes and age structures in the respective countries [10]. These differences imply that system accessibility, ease of use, and national awareness campaigns can shape who reports, what is reported, and how rigorously ADRs are documented.

Notably, there are no special forms for notifying ADRs caused by or associated with drug interactions in Switzerland or Austria (S2 Table). However, both ADR notification forms include dedicated fields for listing co-medications and previous medications, which facilitate the identification and assessment of potential drug interaction-related ADRs by the reporter or the competent authority responsible for drug safety.

A key cross-national difference lies in the assessment of ICSRs in Switzerland, where clinical pharmacologists and clinical pharmacists are integrated into routine hospital care, particularly in university and large cantonal hospitals. This involvement has also recently been identified as an important facilitator of high-quality pharmacovigilance practices for drug manufacturers [18]. These medication specialists contribute critical competencies in assessing and managing drug-related problems through expertise in ADR evaluation, DDI detection, dose adjustment, and formal causality assessments. Although prior studies have demonstrated mixed impacts of such expertise, with improved emergency department outcomes [19] and healthcare costs [20] but limited impact on prescribing quality [21] or hospital admissions [22], specialist involvement on pharmacovigilance reporting quality and DDI recognition remains underexplored.

These structural differences may stem from contrasting approaches to clinical pharmacology training. Switzerland offers a comprehensive six-year specialist training programme in clinical pharmacology and toxicology, with two to three years foundational medical training and three to four years of specialised training in clinical pharmacology and toxicology [23]. In Austria, a sub-specialisation in clinical pharmacology could be achieved by internists, anaesthesiologists, neurologists, psychiatrists, and pharmacologists, but this option was abolished in 2015 during the restructuring of medical specialist training [24]. This policy reversal may have resulted in infrastructure gaps, with departments of clinical pharmacology lacking at all but one Austrian medical university, whereas there are six such departments across Switzerland. In response, the Austrian Pharmacological Society recently applied to the competent authorities to re-establish a specialisation in clinical pharmacology.

In the present study, the Swiss model potentially contributed to the higher pharmacological consistency observed in Swiss reports, reflected in a greater proportion of mechanistically plausible ADRs, such as QT interval prolongation or elevated plasma drug concentrations, and fewer implausible associations. In contrast, Austrian reports more often reflected symptomatic and sometimes inconsistent ADR patterns, including reports linking pantoprazole to gastrointestinal bleeding or risperidone to urinary tract infections. These inconsistencies raise important questions about causality assessment rigor, possible misattribution, and the influence of reporter training or patient reporting biases [29,30]. Patient and consumer reports tend to include more subjective or symptomatic ADRs, sometimes with less pharmacological plausibility, while healthcare professional reports are more likely to be mechanistically consistent and complete in clinical detail [31]. These differences may be influenced by the level of reporter training, the structure of national reporting systems, and the presence or absence of mandatory reporting requirements [25–27]. These findings may point to limitations in pharmacological oversight and warrant confirmative investigation of the value of specialist-led pharmacovigilance approaches in enhancing the interpretability and clinical relevance of spontaneous reports. This suggests a potential opportunity to improve report quality by embedding clinical pharmacologists and pharmacists into Austrian hospital teams and pharmacovigilance oversight. As this monitoring of drug use was already advocated for action in 2012 to integrate these specialists to entangle complex reports [28]. In addition to expert-supported assessment, other measures may strengthen DDI pharmacovigilance systems, including standardized causality assessment frameworks, targeted training for healthcare professionals, user-friendly reporting platforms, integration of electronic clinical decision-support tools, and structured feedback mechanisms for reporters

The observed age distribution differences, with Swiss reports skewing toward older patients, align with the higher prevalence of polypharmacy as a surrogate of higher disease burden and increased susceptibility to pharmacokinetic interactions in the elderly as demonstrated in Swiss cohort studies [29]. Polypharmacy is a well-established risk factor for serious ADRs and medication incidents, with older, multimorbid patients being particularly vulnerable [9,29]. A notable similarity is the prominent involvement of nervous system medications (31–36%) in both countries suggesting that complex psychotropic regimens may represent a particular risk factor for serious drug interactions. This pattern is reflected in a recent analysis of DDIs in US American pharmacovigilance data, ranking psychotropic drugs among the medications most frequently reported in cases of serious adverse events [30]. The higher rate of serious outcomes in polypharmacy cases across both countries reinforces the established link between complex drug regimens and adverse clinical events. However, the association was stronger in Switzerland (Swiss ROR 3.71 vs. Austrian ROR 1.98) and may reflect differences in the application or interpretation of seriousness criteria during reporting, as well as system-level factors influencing reporting behaviour [26].

The present study has certain limitations. First, our analysis utilised pharmacovigilance databases designed primarily for signal detection rather than cross-system comparison or drug interactions. While we employed report characteristics as indicators of system performance, these databases lack standardised metrics specifically developed for evaluating pharmacovigilance system quality, and by extension specialist involvement was not available as a variable. Consequently, observed differences in report quality between countries should not be interpreted as evidence of causality but rather as associations that warrant further investigation. Second, the inherent biases of spontaneous reporting systems, including underreporting, duplicate entries, and reporting stimulated by media or regulatory actions, limit generalisability. Third, causality was not formally assessed and observed drug-ADR associations should be interpreted with caution, particularly in light of inconsistencies found in the Austrian data. Fourth, while completeness scores provided a measure of data quality, these scores do not capture clinical relevance or diagnostic accuracy. The completeness scores observed in both countries (Switzerland 0.47; Austria 0.43) are consistent with a previously reported average of 0.46 in VigiBase^®^ [31]. Finally, spontaneous reporting systems are not suitable for estimating the prevalence or incidence of adverse events or drug interactions among populations exposed to drugs.

A major strength of this study lies in its comprehensive, cross-national analysis of real-world pharmacovigilance data over an extensive period. This approach provides valuable insights into the potential influence of reporting infrastructure and practices on drug interaction surveillance, which lead to differences in coding practices and ADR classifications. Stratification by age group, reporter type, and ADR seriousness offers a nuanced understanding of reporting dynamics. The higher number of reports from Switzerland may reflect differences in reporting activity, reporting culture, or pharmacovigilance system characteristics. These findings provide translatable implications for regulatory bodies and healthcare systems responsible for pharmacovigilance practice internationally.

## Conclusions

This comparative analysis highlights substantial differences in drug interaction reporting and assessment between Switzerland and Austria, possibly driven in part by distinct pharmacovigilance system structures, user engagement, and healthcare professional roles. While Switzerland demonstrated higher reporting volumes and greater pharmacological consistency, Austria showed a broader range of symptomatic and potentially inconsistent ADRs. The structured involvement of clinical pharmacologists and pharmacists in Swiss pharmacovigilance practice appears to support higher report quality and pharmacological plausibility, suggesting a valuable model for enhancing drug safety practices elsewhere. These findings warrant confirmative investigation of the need to enhance harmonisation in expert-supported pharmacovigilance practices, to promote standardised assessments, and to improve user interfaces and training for healthcare professionals. Strengthening these aspects may improve the accuracy and utility of drug interaction reporting, tackling the rising complexity of modern pharmacotherapy, and ultimately contributing to safer prescribing and better patient outcomes.

## Supporting information

S1 Table STROBE statement—checklist of items that should be included in reports of cross-sectional studies. (PDF)

S2 Table Major components of the official notification forms in Switzerland and Austria. (PDF)

S1 File S1-S2 Figs. Temporal trends in drug-interaction involvement by ATC code from 2008–2024. Switzerland, Austria. (PDF)

S2 File S3-S6 Figs. Time series of polypharmacy proportion per serious and non-serious drug interaction cases stratified by country. Switzerland, Austria.
(PDF)

S7 Fig Comedication network analysis stratified by country. We constructed comedication networks by transforming each case report into a list of unique active substances and generating all possible two-drug combinations for that case. Nodes represent individual drugs, and edges represent co-reporting within at least one case; edge weights correspond to the number of shared reports. We calculated node degree (number of unique comedication partners) and node strength (weighted degree based on edge counts). For visualization, we plotted the top-degree drugs using horizontal bar plots with an adjacent table summarizing degree, weighted degree, and number of cases in which each drug was reported.
(PDF)

## Abbreviations

ADR: Adverse drug reaction
ATC: Anatomical-therapeutic-chemical
BASG: Federal Office for Safety in Health Care
CI: Confidence interval
CV: Coefficient of Variance
DDI: Drug-drug interaction
ElViS: Electronic Vigilance System
ICSR: Individual case safety report
IQR: Interquartile range
MedDRA: Medical Dictionary for Regulatory Activities
ROR: Reporting odds ratio
rSD: Rolling standard deviation
SD: Standard deviation
STROBE: Strengthening the Reporting of Observational Studies in Epidemiology
UMC: Uppsala Monitoring Centre
