# Supplementary material for: Cross-national disparities in drug interaction reporting and assessment between Switzerland and Austria: A retrospective, registry-based study using pharmacovigilance data
Source: PLoS One. 2026 Aug 21;21(8):e0356774. doi: 10.1371/journal.pone.0356774 (PMC13496446; doi:10.1371/journal.pone.0356774)
Supplement: Supplementary file 2 [file pone.0356774.s002.pdf]

S2 Table Major components of the official notification forms in Switzerland and Austria.

**Country**<br>**Competent authority, form**	Switzerland<br>Swissmedic ElViS	Austria<br>BASG online form
**Accessibility**	Registration required	Identification of the reporting person, easily accessible for lay persons
**Structure and contents**	Patient initials, date of birth, age at the beginning of the ADR, ADR severity, category, sequalae, examination results, suspected drug (1 at least), risk factors: pregnancy, alcohol, smoking, ADR: judgement commentary, attachment, reporting person, receiver	Reporting person, function, name, contact data, patient DOB, sex, mandatory, state (province), ADR list, drug, start of treatment, end of therapy, trade name (mandatory), lot no, causality, previous diseases, underlying disease, course of disease, previously applied drug relevant for the ADR
**Mandatory fields**	Patient DOB, sex, age group, beginning of ADR, list ADRs, sequalae, suspected drugs	Reporting person, function, name, contact data, patient DOB, sex, beginning of ADR, ADRs select from list 15 options including “ADR not mentioned above”, causality, trade name, data safety declaration
**Comedication**	Comedication (asked together with the suspected medication) more explicit, but no mandatory filed	Further, previously applied drug that are relevant for the ADR, (no explicit question for simultaneous taken comedication)
**DDI**	No explicit question for DDI	No explicit question for DDI

Abbreviations: *ADR, Adverse Drug Reaction; BASG, Bundesamt für Sicherheit im Gesundheitswesen [engl.: Austrian Federal Office for Safety in Health Care]; DDI, Drug-Drug Interaction; DOB, Date of Birth; ElViS, Swiss Electronic Vigilance System.*
