# Supplementary material for: Cross-national disparities in drug interaction reporting and assessment between Switzerland and Austria: A retrospective, registry-based study using pharmacovigilance data
Source: PLoS One. 2026 Aug 21;21(8):e0356774. doi: 10.1371/journal.pone.0356774 (PMC13496446; doi:10.1371/journal.pone.0356774)

Temporal trends in drug-interaction involvement by ATC code from 2008-2024

Switzerland

S1 Fig

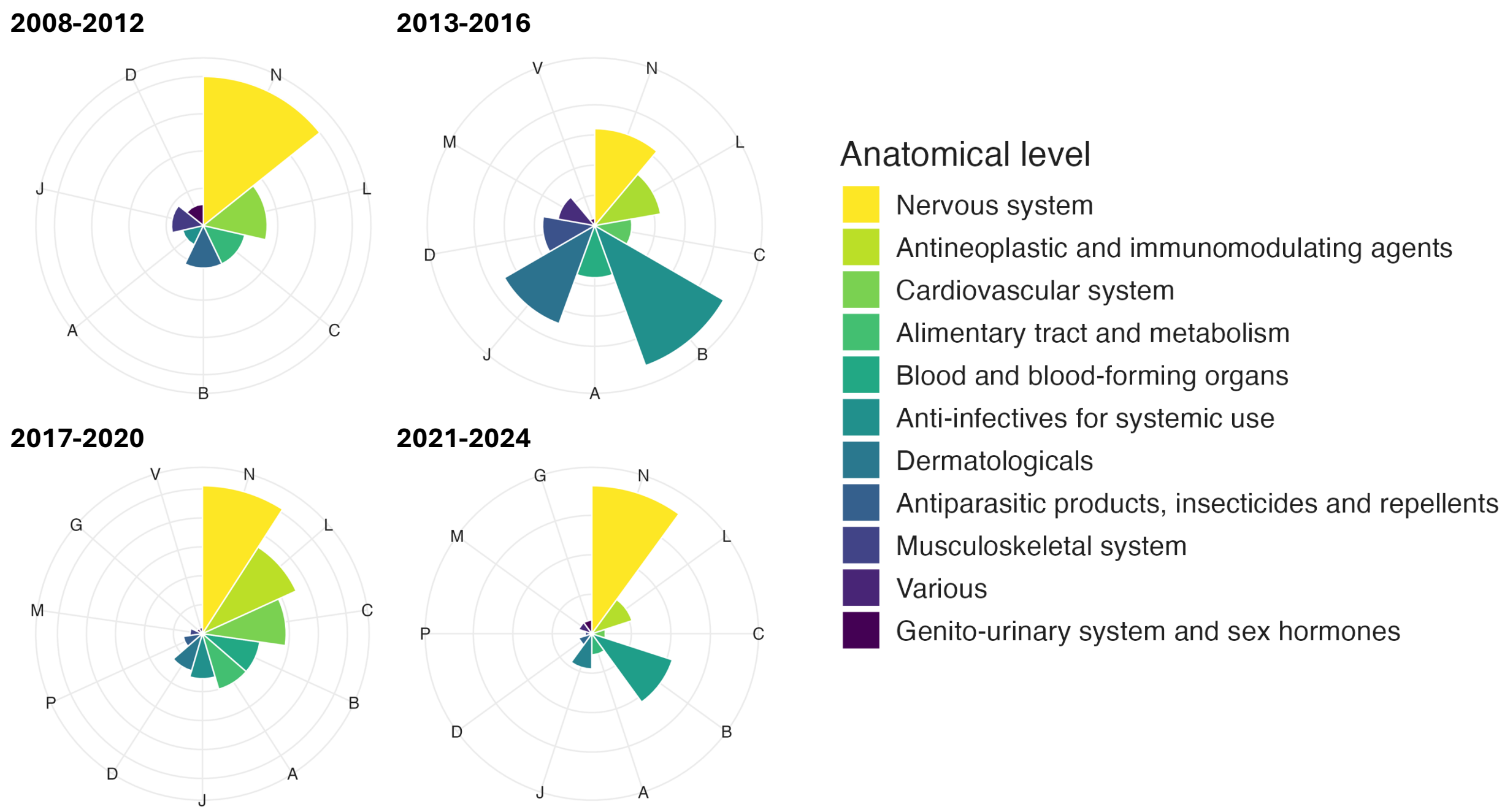

Austria

S2 Fig

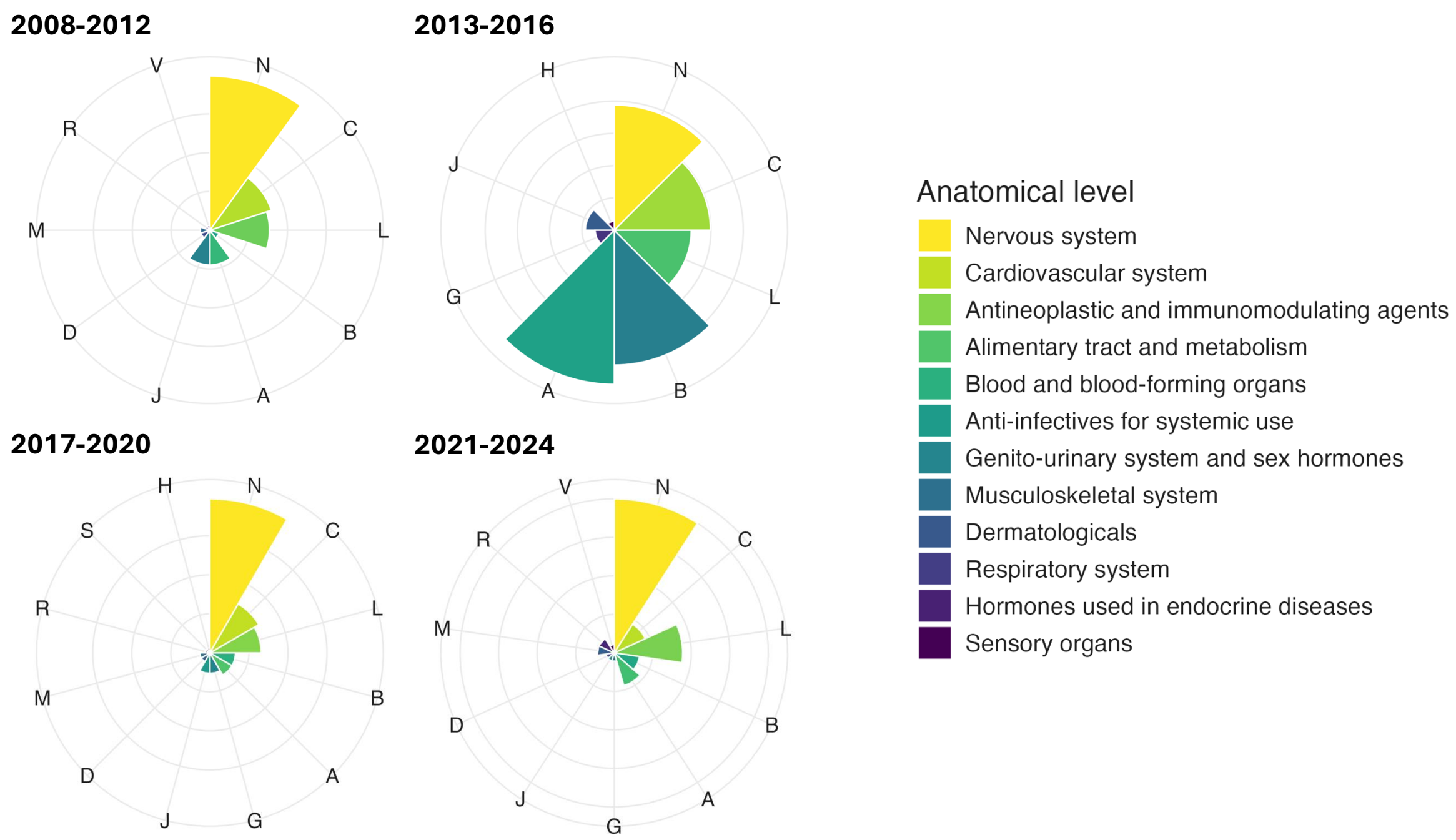

Supplement: Supplementary file 3 [file pone.0356774.s003.pdf]
