# Supplementary material for: Cross-national disparities in drug interaction reporting and assessment between Switzerland and Austria: A retrospective, registry-based study using pharmacovigilance data
Source: PLoS One. 2026 Aug 21;21(8):e0356774. doi: 10.1371/journal.pone.0356774 (PMC13496446; doi:10.1371/journal.pone.0356774)
Supplement: Supplementary file 4 [file pone.0356774.s004.pdf]

Time series of polypharmacy proportion per serious and non-serious drug interaction cases stratified by country

Switzerland

S3 Fig

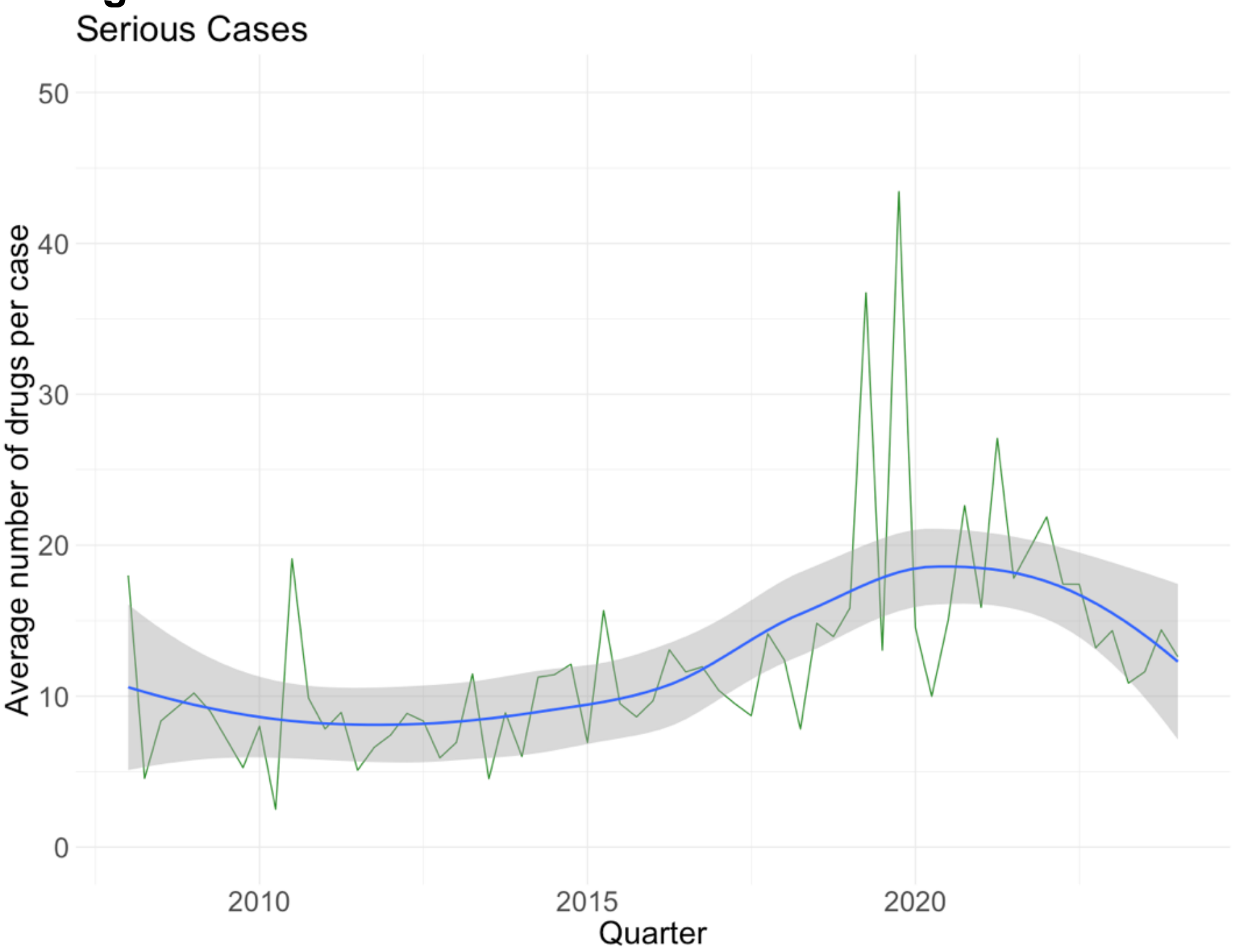

Polypharmacy proportion = 68.7%

S4 Fig

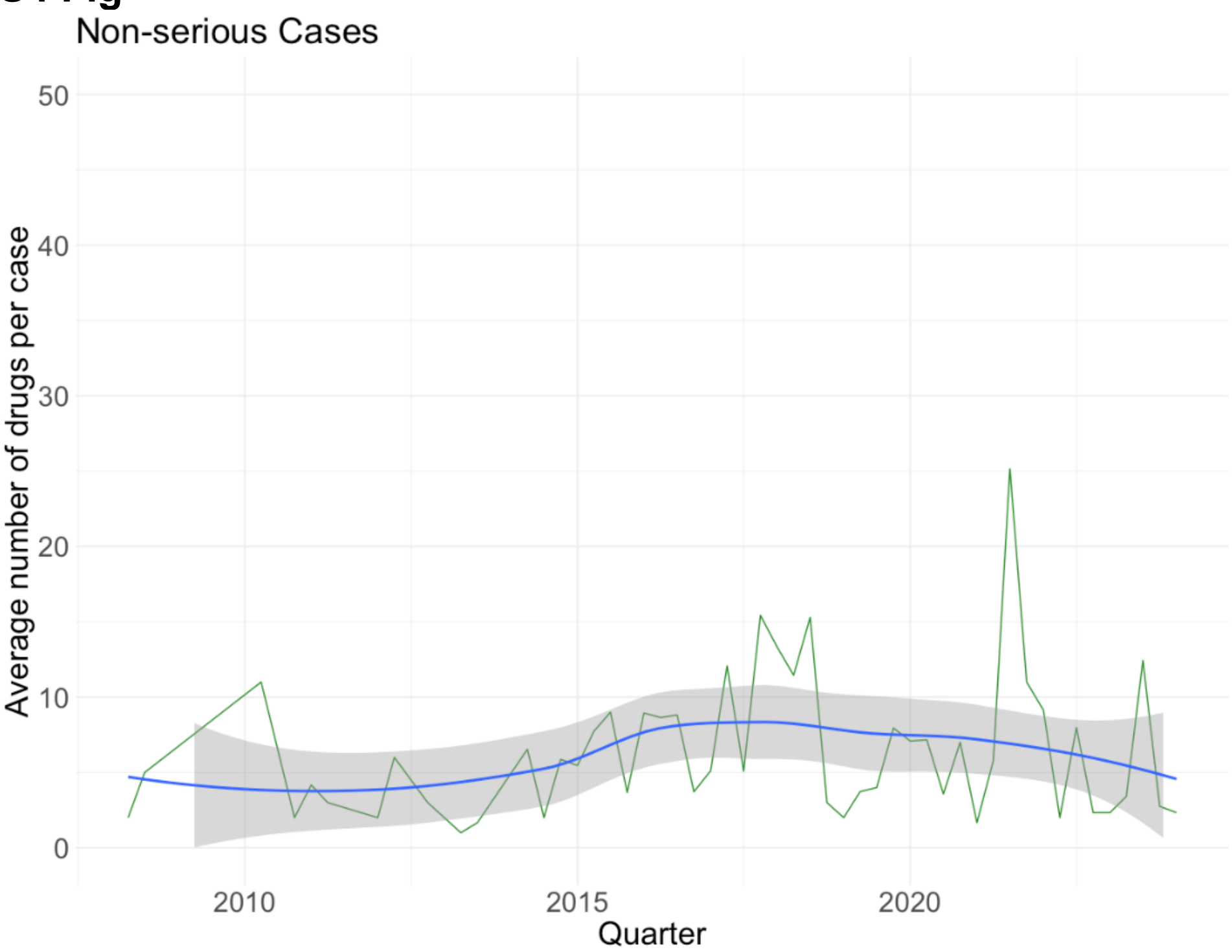

Polypharmacy proportion = 30.3%

Austria

S5 Fig

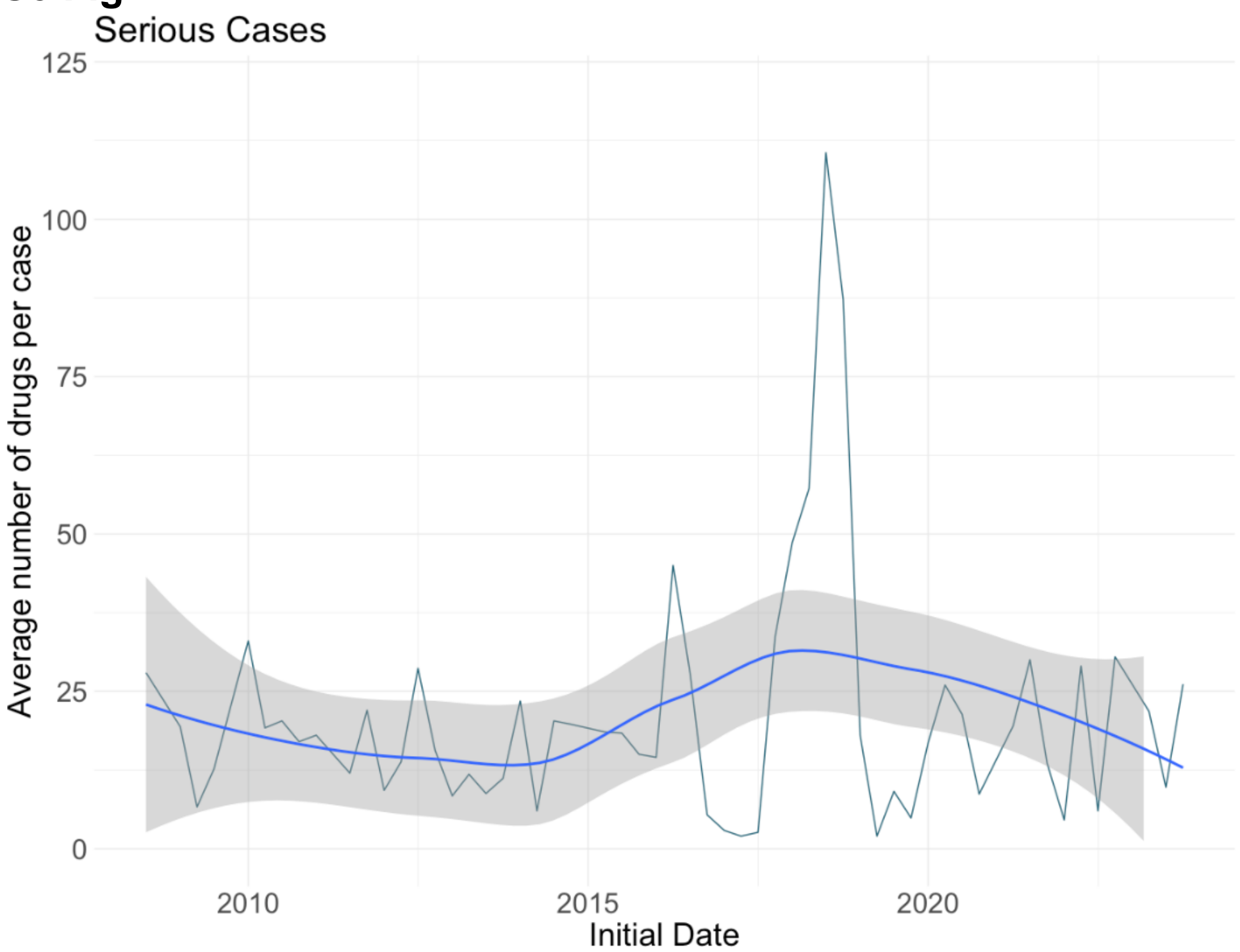

Polypharmacy proportion = 71.4%

S6 Fig

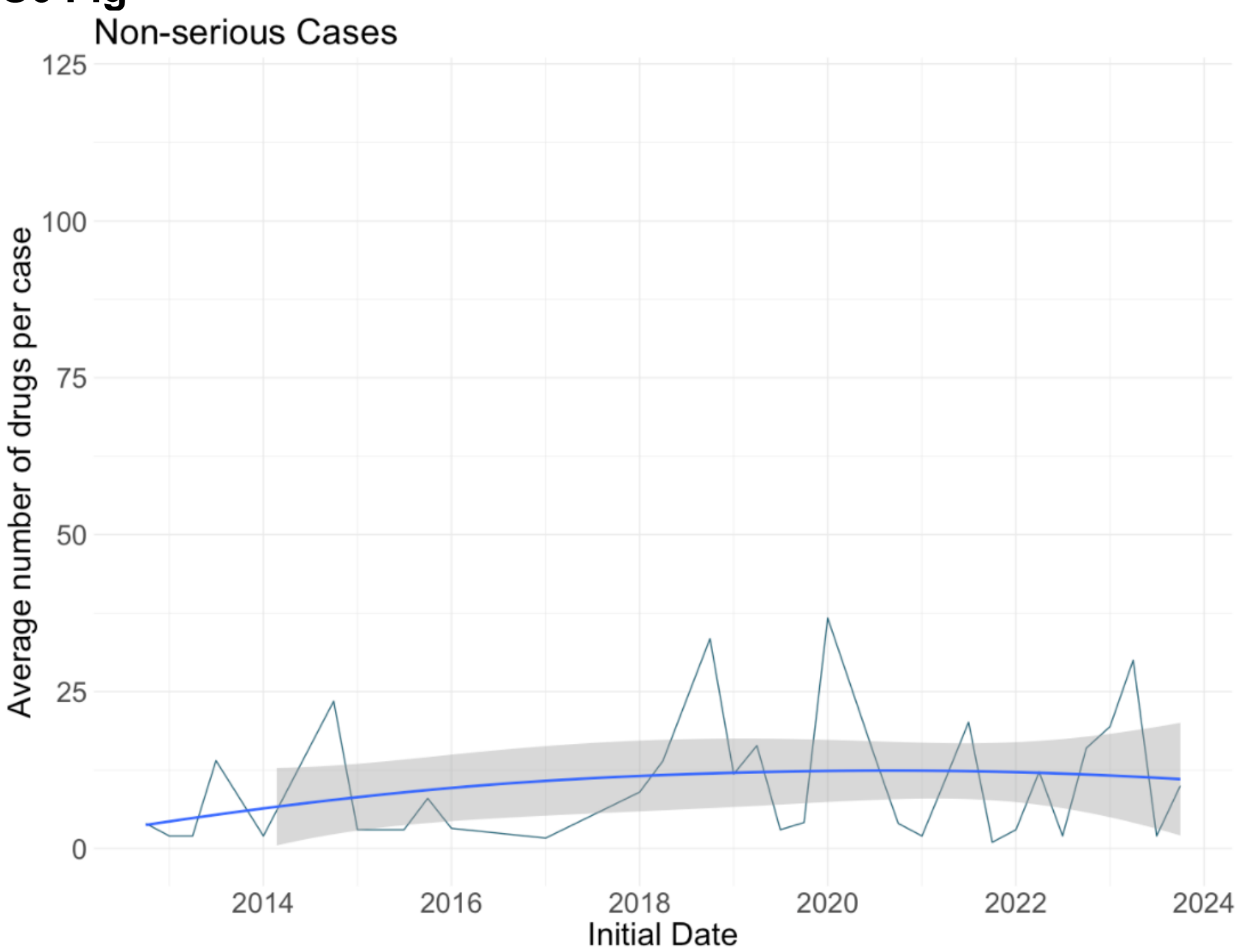

Polypharmacy proportion = 46.3%
