# Supplementary material for: Cross-national disparities in drug interaction reporting and assessment between Switzerland and Austria: A retrospective, registry-based study using pharmacovigilance data
Source: PLoS One. 2026 Aug 21;21(8):e0356774. doi: 10.1371/journal.pone.0356774 (PMC13496446; doi:10.1371/journal.pone.0356774)
Supplement: Supplementary file 5 [file pone.0356774.s005.pdf]

S7 Fig. Comedication network analysis stratified by country. We constructed comedication networks by transforming each case report into a list of unique active substances and generating all possible two-drug combinations for that case. Nodes represent individual drugs, and edges represent co-reporting within at least one case; edge weights correspond to the number of shared reports. We calculated node degree (number of unique comedication partners) and node strength (weighted degree based on edge counts). For visualization, we plotted the top-degree drugs using horizontal bar plots with an adjacent table summarizing degree, weighted degree, and number of cases in which each drug was reported.

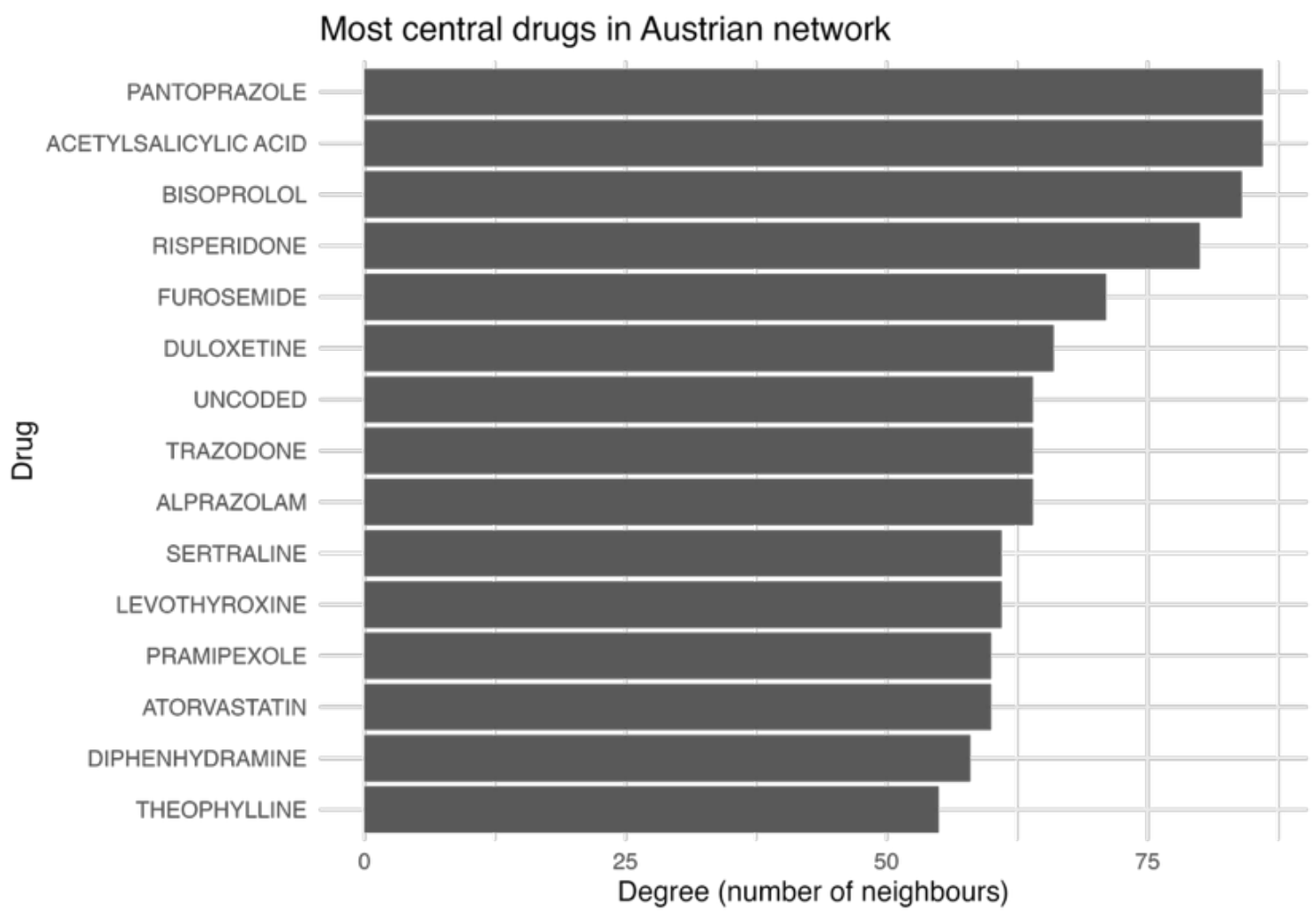

Drug	Degree	Strength	# cases
ACETYLSALICYLIC ACID	86	339	53
PANTOPRAZOLE	86	256	42
BISOPROLOL	84	350	37
RISPERIDONE	80	302	31
FUROSEMIDE	71	274	24
DULOXETINE	66	258	28
ALPRAZOLAM	64	256	21
TRAZODONE	64	226	41
UNCODED	64	194	30
LEVOTHYROXINE	61	237	19
SERTRALINE	61	228	35
ATORVASTATIN	60	242	22
PRAMIPEXOLE	60	204	11
DIPHENHYDRAMINE	58	156	5
THEOPHYLLINE	55	215	16

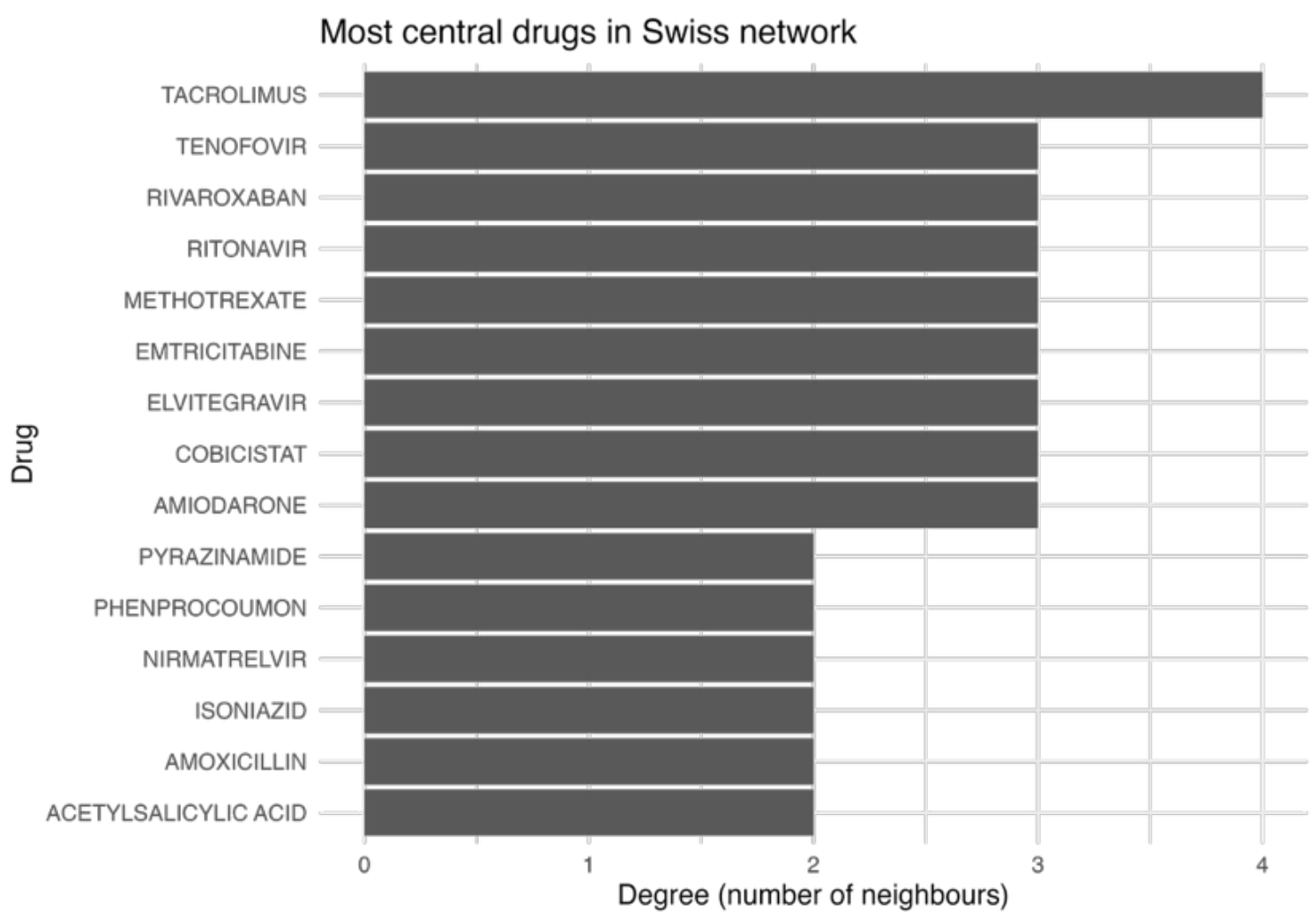

Drug	Degree	Strength	# cases
TACROLIMUS	4	17	21
AMIODARONE	3	16	25
COBICISTAT	3	12	4
ELVITEGRAVIR	3	12	4
EMTRICITABINE	3	14	6
METHOTREXATE	3	11	21
RITONAVIR	3	13	11
RIVAROXABAN	3	16	35
TENOFOVIR	3	14	6
ACETYLSALICYLIC ACID	2	13	22
AMOXICILLIN	2	6	5
ISONIAZID	2	8	5
NIRMATRELVIR	2	9	5
PHENPROCOUMON	2	6	22
PYRAZINAMIDE	2	8	4
